# Supplementary material for: The natural function of the malaria parasite’s chloroquine resistance transporter
Source: Nat Commun. 2020 Aug 6;11:3922. doi: 10.1038/s41467-020-17781-6 (PMC7413254; doi:10.1038/s41467-020-17781-6)
Supplement: Supplementary file 1 — Supplementary Information [file 41467_2020_17781_MOESM1_ESM.pdf]

Supplementary Information

The natural function of the malaria parasite's chloroquine resistance transporter

Shafik *et al*.

Supplementary Note 1

Detecting *cis*-inhibition of [^{3}H]CQ uptake into oocytes expressing PfCRT. As mentioned in the main text, CQ transport via wild-type PfCRT (PfCRT^{3D7}) is not detected under normal conditions. This has been observed in both the *Xenopus* oocyte system (using [^{3}H]CQ transport assays[1, 2]) and in live parasites (using the H^+-efflux assay[3-5]). The low level of [^{3}H]CQ accumulation measured in oocytes expressing PfCRT^{3D7}, which is the same level as that present in the non-expressing oocytes, is due to the simple diffusion of the neutral species of the drug into the oocyte[1]. This component of uptake is the background accumulation of [^{3}H]CQ that occurs independently of PfCRT. The process of simple diffusion is not saturable, nor is it inhibited by other solutes. Hence, when the unlabelled solutes are screened for the ability to *cis*-inhibit the uptake of [^{3}H]CQ into oocytes, all are without effect in both the non-expressing and PfCRT^{3D7}-expressing oocytes (Fig. 1b,c,e, Supplementary Fig. 1a,b, and Supplementary Data 1,2) because [^{3}H]CQ enters these oocytes only via simple diffusion. By contrast, the uptake of [^{3}H]CQ via the two mutant isoforms of PfCRT (PfCRT^{Ecu1110} and PfCRT^{Dd2}) can be *cis*-inhibited by the binding of unlabelled inhibitors or substrates to the transporter.

It should be noted, however, that when PfCRT^{3D7} is *trans*-stimulated by millimolar concentrations of one of its unlabelled native substrates (see Supplementary Note 2, Fig. 2, 5d, and Supplementary Data 1,2), a modest but significant rate of [^{3}H]CQ transport is observed above the background rate of diffusion. These results show that whilst PfCRT^{3D7} mediates little or no [^{3}H]CQ transport under normal conditions, strong *trans*-stimulation of the protein produces a [^{3}H]CQ transport signal that is readily detected and reproduced.

The *cis*-inhibition assay measures the ability of an unlabelled solute to compete with the binding of [^{3}H]CQ to PfCRT^{Ecu1110} and PfCRT^{Dd2}. Inhibition is detected as a decrease in the rate of [^{3}H]CQ transport relative to the positive control (which is [^{3}H]CQ transport via PfCRT^{Ecu1110} or PfCRT^{Dd2} in the absence of an unlabelled test solute). The high signal-to-background ratio for CQ uptake in the PfCRT oocyte system, as well as the reproducibility of the assay, enables relatively modest decreases in the [^{3}H]CQ transport rate to be quantified. This, together with the observation that the IC_{50} values for the inhibition of [^{3}H]CQ transport by known substrates of PfCRT^{Dd2} (e.g. quinine, methylene blue, quinacrine, verapamil, and amantadine)

range from 13 μM to 1.3 mM[6-8], supported testing the unlabelled solutes for *cis*-inhibition at 0.5 – 2 mM.

Supplementary Note 2

Identification of the native substrates of PfCRT using a *trans*-stimulation assay. We employed a *trans*-stimulation assay in conjunction with the oocyte expression system to distinguish between inhibitors and substrates of PfCRT and, once we had identified peptides as the transporter's natural substrates, to determine the range of peptides transported via PfCRT (Fig. 2b-g, 5d, Supplementary Fig. 1, and Supplementary Data 1,2).

Trans-stimulation is a phenomenon exhibited by many carrier-type transporters (i.e. the class of membrane transport protein to which PfCRT belongs[9]), whereby the reorientation of the binding site between the two faces of the membrane occurs more rapidly when it is occupied by a substrate than when it is empty. This property means that the PfCRT-mediated uptake of [^{3}H]CQ from the extracellular solution will increase when an unlabelled substrate is added to the cytosolic face of the membrane (Fig. 2a), but not when the unlabelled solute is not translocated by PfCRT[7]. Hence, by measuring the ability of unlabelled solutes to *trans*-stimulate the transport of a labelled substrate, the substrate-specificity of a transporter can be elucidated without the prohibitive expense of purchasing multiple radiolabelled solutes.

Another important characteristic of carrier-type transporters is that any solute shown to be transported in one direction across the membrane can also be transported in the opposite direction. This is a macroscopic consequence of the principle of microscopic reversibility (an adjunct to the laws of thermodynamics). That is, there is no right or wrong direction and the activity of the protein can be assessed by measuring transport in either direction[10-13]. In this study, we have shown peptide/peptide mimic transport via PfCRT in both directions. For example, the experiments in which the peptide mimic saquinavir was used to *trans*-stimulate the PfCRT-mediated efflux of [^{3}H]VF-6 from the oocyte is an example of transport occurring in the direction that equates to the efflux of the peptide mimic from the parasite's DV. It should be noted, however, that the *trans*-stimulation assay does not reveal the net direction in which substrate transport occurs *in vivo*. In any case, the large outwardly-directed gradients

across the DV membrane for both protons and peptides would enable little, if any, transport of peptides into the vacuole.

In the experiments giving rise to the datasets presented in Fig. 2b-g, 5d, Supplementary Fig. 1, and Supplementary Data 1,2, the test solute was injected into the oocyte immediately prior to the commencement of the assay. The approximate intracellular concentrations of the test solutes were 5 and 45 mM (or 35 mM if solubility in the aqueous buffer was limiting), and the injection controls were the buffer-only and LH treatments[7]. The negative controls were non-expressing oocytes and those expressing an unrelated *P. falciparum* transporter, the nucleoside transporter 1 (PfNT1[14, 15]). The latter demonstrates that the expression of a similarly-sized transporter in *Xenopus* oocytes does not affect the ability of the oocyte membrane to reseal following the injection of a test solute[7].

Trans-stimulation has occurred if there is an increase in the transport rate above that measured in the relevant positive control. The extent to which a carrier can be *trans*-stimulated above its zero-*trans* rate of transport will depend on factors that are inherent to its structure-function, such as the activation energy (E_a) for substrate translocation[16]. For some transporters, *trans*-stimulation increases the transport rate by several fold, whereas other carriers exhibit more modest increases – for example, the *trans*-stimulated rate may only be 20-50% above the zero-*trans* rate. Hence, *trans*-stimulation can be more difficult to quantify than *cis*-inhibition, especially if the unlabelled test solute is a poor substrate and/or the re-orientation of the empty carrier is not a substantially rate-limiting step in the translocation cycle. We therefore used high concentrations of the test solutes in the *trans*-stimulation assays (5 mM and 35/45 mM) to optimise the detection of increases in the rate of [^{3}H]CQ transport.

These two solute concentrations are also approximations of the levels to which peptides – and other metabolites/catabolites – could be expected to accumulate within the DV. For example, during the 48 hours it grows and replicates within an erythrocyte, the malaria parasite consumes 50-80% of the host cell's haemoglobin. The intraerythrocytic concentration of haemoglobin is ~5 mM and its quaternary structure consists of four haem molecules per haemoglobin tetramer[17]. Some of the peptide sequences occur twice in the haemoglobin tetramer (e.g. VF-6), others occur six (DL and HL) or eight (VD) times. Several other factors need to be considered when estimating the level to which a given peptide is likely to

be present within the vacuole. First, the volume of the erythrocyte is ~75 fl[18] and the volume of the DV is estimated to reach ~7 fl during the trophozoite stage of the parasite[18, 19]. Secondly, haemoglobin digestion begins in the ring stage and continues throughout the trophozoite stage (to at least 32 hours post-invasion), and over this period PfCRT will be working to remove these peptides from the vacuole. That is, only a fraction of the peptide generated by the degradation of the host's haemoglobin will be present in the vacuole at any one time.

If 50-80% of host's haemoglobin was degraded into peptides, and none of these peptides were exported from the vacuole or promiscuously degraded, the concentration of VF-6 would be 27 mM (5 mM $\times$ 75 fl $\times$ 0.5 $\div$ 7 fl) to 43 mM (5 mM $\times$ 75 fl $\times$ 0.8 $\div$ 7 fl). However, the actual concentration will be much lower than these values because haemoglobin digestion takes a number of hours to complete, and the resulting peptides are constantly being exported from the vacuole. That said, the digestion of haemoglobin (and of other host cell proteins) will generate many different species of peptide substrates for PfCRT; all of these will be present at millimolar levels and all will compete for translocation via PfCRT. Under such highly competitive and saturating conditions, a poor substrate of the carrier will undergo negligible transport. Hence, any unlabelled solute that fails to elicit a *trans*-stimulation signal when present at 35/45 mM is not a physiologically-relevant substrate of the carrier.

Supplementary Note 3

Rationale for including the solutes with little or no *cis*-inhibitory activity against PfCRT in the *trans*-stimulation assay. The extent to which a given unlabelled solute *cis*-inhibits the translocation of a radiolabelled substrate is not always an accurate predictor of (1) whether the unlabelled solute is itself a substrate of the transporter or (2) the extent to which it may *trans*-stimulate the transport of the radiolabelled substrate.

For example, a solute can be a potent inhibitor, but a poor substrate, of a transporter – and such cases have previously been described for PfCRT[6-8]. Since a poor substrate will also be a poor *trans*-stimulator (because the low rate of transport results in a correspondingly low rate of carrier reorientation),

its potent *cis*-inhibition activity does not translate into a strong *trans*-stimulatory effect. Furthermore, if the transporter has a poly-specific binding cavity (as does PfCRT[6]), there can be cases where the unlabelled solute is a relatively modest *cis*-inhibitor of the transport of another (radiolabelled) substrate, but is nonetheless a strong substrate of the carrier. Such a case has been demonstrated for PfCRT[8]. Given the potential for there to be a disconnect between the magnitude of *cis*-inhibition exerted by a solute and its ability to *trans*-stimulate the carrier, we tested all of the unlabelled solutes for both *cis*-inhibitory and *trans*-stimulatory activities.

Supplementary Note 4

The effect of the oocyte membrane potential on transport via PfCRT. We have previously shown that CQ uptake via $PfCRT^{Dd2}$ decreases when the oocyte membrane potential is depolarised[1], which is consistent with the translocation step involving the movement of a net positive charge – CQ and at least one proton – into the oocyte. The dataset presented in Supplementary Fig. 2g confirms this finding and characterises the effect in greater detail – i.e. numerous measurements of [^{3}H]CQ influx were made between -80 mV and +80 mV.

Application of a similar assay to the efflux of [^{3}H]VF-6 from the oocyte revealed that the rate of PfCRT-mediated transport steadily decreases as the oocyte membrane potential becomes more positive. As mentioned in the main text, peptide transport via PfCRT is dependent both on protons and a second solute that remains to be identified, but which is naturally present within the oocyte. All three co-substrates must be present on the same side of the membrane for the transport of host-derived peptides to occur. We therefore co-injected [^{3}H]VF-6 and protons into the oocyte and measured peptide efflux as the potential was varied between -80 mV and +80 mV. The resulting dataset (Fig. 3d) indicates that the efflux of [^{3}H]VF-6 and its co-substrates involves the movement of a net negative-charge out of the oocyte. Given that the peptide is negatively-charged and is translocated in symport with a proton, one possible scenario would be that the second co-transported ion is also negatively-charged (as this would produce a net negative charge).

In this regard, we note that several substrates of PfCRT – e.g. CQ, quinine, quinidine, quinacrine, methylene blue, saquinavir, amantadine, verapamil, and the endomorphin peptide YPWF-NH_2 – only require protons to undergo translocation[1, 6-8]. That is, their transport is decoupled from the second ion, thus enabling measurements of their uptake from the extracellular solution into the oocyte. The decoupling of some substrates, but not others, from the translocation of a co-substrate is a known phenomenon in carrier-type transporters. For example, the mammalian System y^+L mediates the Na^+-dependent transport of neutral amino acids and the Na^+-independent transport of cationic amino acids[20, 21], and a bacterial Nramp transporter mediates the H^+-dependent transport of Mn^{2+} as well as the H^+-independent transport of Cd^{2+}[22].

In the table shown below, we identify three features that differ (for the most part) between the PfCRT substrates that require the second co-substrate and those that do not. However, given that saquinavir was the only peptide mimic or modified peptide to be included in the assay that detected decoupling from the second co-substrate, there is a limit to what can be extrapolated about this phenomenon from our current knowledge.

**Feature**	**Solutes transported with 2nd co-substrate** (host-derived peptides)	**Solutes transported without 2nd co-substrate** (chloroquine, quinine, quinidine, quinacrine, methylene blue, saquinavir, amantadine, verapamil, & YPWF-NH_2)
Zwitterion	**YES**	**NO** except for YPWF-NH_2
Complete peptide backbone	**YES**	**NO** except for YPWF-NH_2
Quinoline moiety	**NO**	**YES** except for YPWF-NH_2, amantadine, and verapamil

It is worth noting that the three exceptions listed in the far-right column – YPWF-NH_2, amantadine, and verapamil – are poor substrates of $PfCRT^{Dd2}$ in the oocyte system[1, 6, 7]. Indeed, of all the substrates characterised in this system to date, YPWF-NH_2, amantadine, and verapamil produced by far the lowest signal-to-background ratios for transport via $PfCRT^{Dd2}$. Hence, it may well be that the translocation rates of these three solutes would increase in the presence of the second co-substrate. In which case, their transport may be considered to be mostly dependent on the second co-substrate, with a degree of

‘slippage’ occurring. Slippage is defined as the translocation of a substrate without one or more of its usual co-substrates. This property has been observed in a number of carriers[23-25].

These caveats aside, it is possible that one or more of the three features we have identified – zwitterion, complete peptide backbone, and quinoline moiety – determine whether or not the transport of a solute via PfCRT requires the second co-substrate. Further characterisation of this phenomenon in the oocyte system, such as the testing of a broader range of drugs, peptide mimics, and modified peptides for the ability to be transported via PfCRT without the second co-substrate, will be necessary to distinguish between these possibilities.

Supplementary Note 5

The addition of polypeptide sequences to the termini of PfCRT abolishes VF-6 transport activity: We found that the addition of a tag to PfCRT – regardless of polypeptide sequence, length, or it being at the N- or C-terminus – abolishes VF-6 transport activity (Fig. 4c, right panel). That is, regardless of whether PfCRT is tagged with 1xmyc, 3xmyc, 4xmyc, or 1xHA, in all cases the modified proteins fail to transport the peptide. By contrast, only one of the tagged $PfCRT^{Dd2}$ proteins is unable to transport CQ ($PfCRT^{Dd2}$ carrying a C-terminal 3xmyc tag; Fig. 4c, left panel). The other four variants of $PfCRT^{Dd2}$ retain all or most of their CQ transport activity.

These findings suggest that the extension of either termini prevents the peptide from entering the binding cavity and/or from binding therein. This could occur because the termini are located near the substrate-binding cavity, and the added polypeptide juts either into, or across the top of, the cavity. Alternatively, the effect could be indirect, whereby the terminus is not proximal to the cavity and the addition of the polypeptide induces a conformational change in PfCRT that prevents the peptide from accessing, or binding to, the cavity. The observation that all bar one of the tagged $PfCRT^{Dd2}$ proteins retain the ability to translocate CQ suggests that the drug, which is much smaller than the peptide, is able to access, and bind to, the altered cavity. It is not clear why the C-terminal 3xmyc tag obstructs CQ transport; it appears that there is a subtle, but functionally significant, difference in the conformation of the

transporter between the version carrying the 3xmyc tag and those carrying 1xmyc, 4xmyc, or 1xHA. Further insight into this phenomenon could be gained from structural determinations of PfCRT. However, only one structure of PfCRT has been published thus far[26], and it did not encompass the N- or C-terminus of the transporter.

Supplementary Note 6

The correlation between the accumulation within CQ-resistant parasites of peptides 4-11 residues in length and their capacity to *trans*-stimulate PfCRT. Our datasets reveal that wild-type PfCRT transports a broad range of host-derived peptides from the parasite's DV, and that PfCRT^{Ecu1110} and PfCRT^{Dd2} have reduced capacities for peptide transport – both in terms of maximum velocity of transport and the range of peptides translocated. Given that the accumulation of host-derived peptides within the CQ-resistant lines could be explained by the reduced capacities of the mutant PfCRT isoforms for peptide efflux, we investigated the relationship between the peptides found to accumulate within the C4^{Dd2} line and those that had been identified as substrates of PfCRT^{3D7} in the *Xenopus* oocyte system (Fig. 7). It was important to use the PfCRT^{3D7} *trans*-stimulation dataset, rather than that generated for PfCRT^{Dd2}, because the peptides most likely to accumulate in the C4^{Dd2} parasite lines will include those that are very poor substrates of (or no longer transported by) PfCRT^{Dd2}. This comparison identified a positive correlation between the ability of a given host-derived peptide to serve as a substrate of wild-type PfCRT and an increase in its accumulation within the C4^{Dd2} line (consistent with the reduced capacity of PfCRT^{Dd2} for exporting said peptides out of the DV) (Fig. 7).

There are two types of exceptions to this relationship. The first is typified by PL-4, which did not *trans*-stimulate PfCRT and yet was found to accumulate within the C6^{7G8} and C4^{Dd2} parasites. Given that PS-5, DS-7, and VL-11 (all peptides immediately upstream of PL-4) are substrates of PfCRT^{3D7} and also accumulate within the CQ-resistant lines, it is likely that the elevated levels of PL-4 are due to the build-up and promiscuous degradation of upstream peptides within the DV. The second outlier is YF-5, which was a modest *trans*-stimulator of all three PfCRT isoforms in the oocyte system and also a substrate *in situ*, but

does not accumulate within the C6^{7G8} and C4^{Dd2} parasites. However, at least one peptide within the same degradation cascade – PS-6 – *trans*-stimulates PfCRT^{3D7} and also accumulates within the CQ-resistant lines. It is possible that competition with the other peptide substrates for transport via PfCRT^{3D7} results in a rate of YF-5 efflux that is not greater than those achieved by the mutant transporters (which accept a narrower range of peptides).

Supplementary Figures

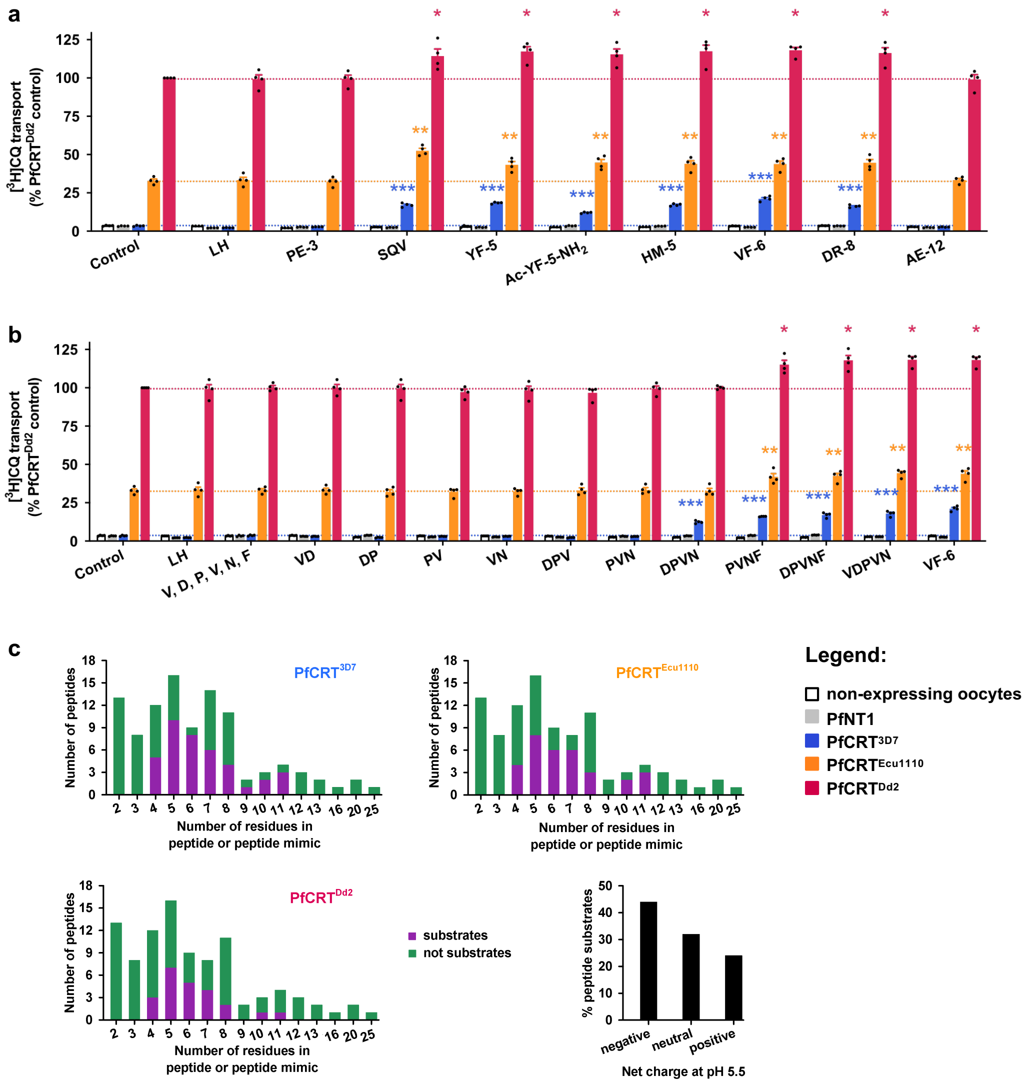

Supplementary Fig. 1. PfCRT is *trans*-stimulated by peptides containing 4-11 residues in *Xenopus* oocytes. **a**, *Trans*-stimulation of [^{3}H]CQ transport via PfCRT by a subset of host-derived peptides and peptide mimics (45 mM). **b**, [^{3}H]CQ transport via PfCRT^{3D7} is *trans*-stimulated by 4-5 residue fragments of VF-6, whereas amino acids, dipeptides, and tripeptides are without effect. **c**, PfCRT^{3D7} is *trans*-stimulated by a broader range of peptides than PfCRT^{Ecu1110} and PfCRT^{Dd2}. The data are the mean of n = 4 independent experiments (each yielding similar results and overlaid as individual data points in **a** and **b**) and the error is the SEM. Where not visible, the error bars fall within the symbols. The asterisks denote a significant difference from the relevant PfCRT^{Dd2} (red asterisks), PfCRT^{Ecu1110} (orange asterisks), or PfCRT^{3D7} (blue asterisks) buffer-injected control; *$P < 0.05$, **$P < 0.01$, ***$P < 0.001$, ns not significant (one-way ANOVA). SQV, saquinavir. The source datasets are provided as a Source Data file.

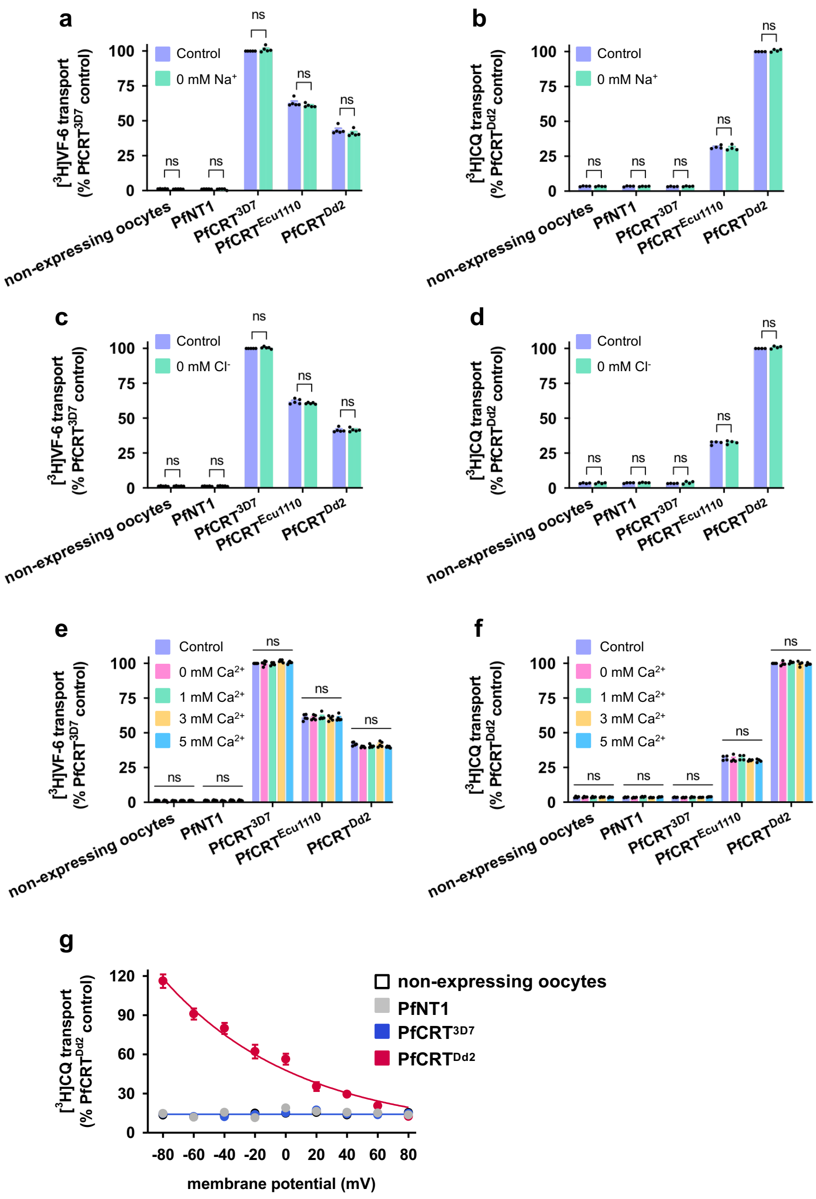

Supplementary Fig. 2. The effects of Na^+, Cl^-, Ca^{2+}, and the membrane potential on transport via PfCRT in *Xenopus* oocytes. a-f, [^{3}H]VF-6 transport is not affected by the removal of Na^+ (**a**) or Cl^- (**c**) from the injection buffer, nor by changes in the concentration of Ca^{2+} (**e**). [^{3}H]CQ transport is likewise unaffected by the removal of Na^+ (**b**) or Cl^- (**d**) from the reaction buffer and remains unchanged when the Ca^{2+} concentration is varied (**f**). **g**, The rate of [^{3}H]CQ uptake via PfCRT steadily decreases as the membrane potential becomes more positive. The data in panels **a**, **c**, and **e** are the mean of n = 5 independent experiments and the data in panels **b**, **d**, **f**, and **g** are the mean of n = 4 independent experiments (each yielding similar results and overlaid as individual data points in **e**-**f**). The error is the SEM; where not visible, the error bars fall within the symbols. ns denotes no significant difference from the relevant control treatment ($P > 0.05$; one-way ANOVA). The source datasets are provided as a Source Data file.

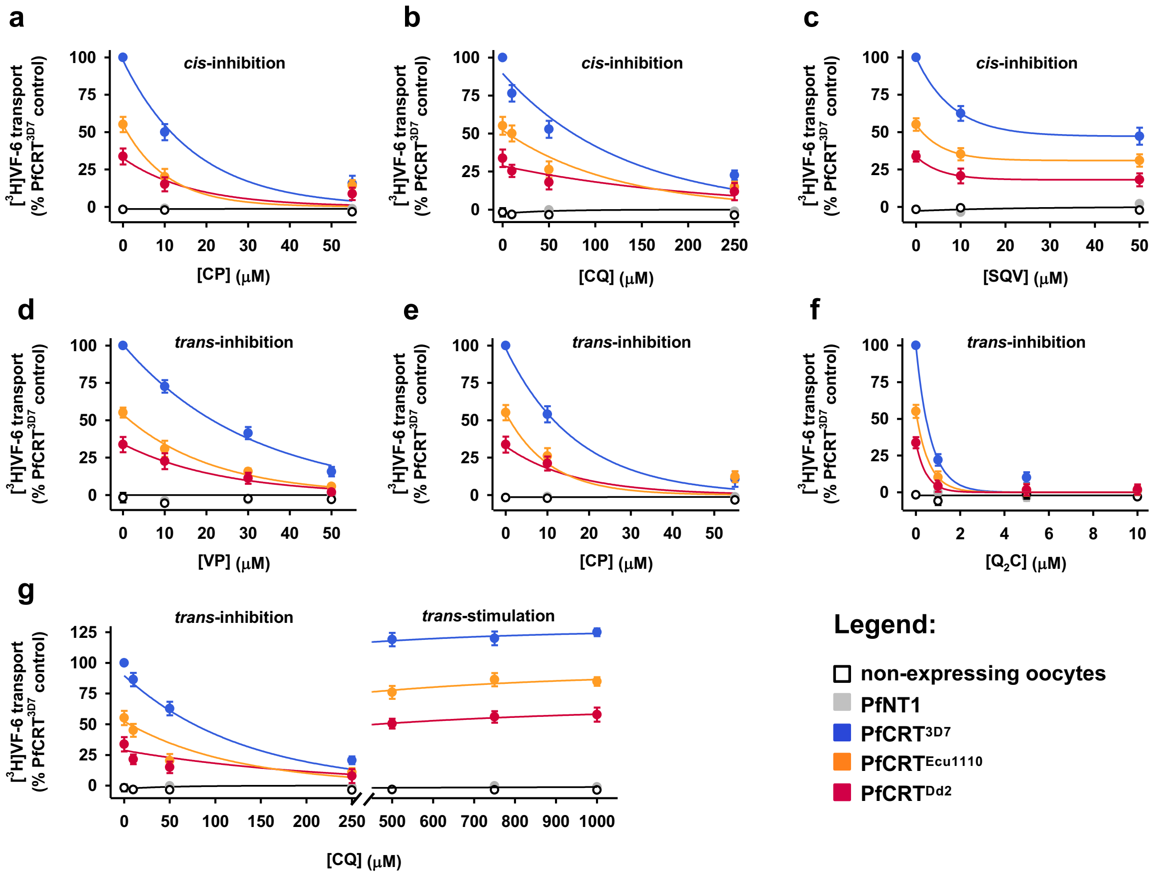

h

	Cis-inhibition IC_{50}s (μM)			*Trans*-inhibition IC_{50}s (μM)		
	PfCRT^{3D7}	PfCRT^{Ecu1110}	PfCRT^{Dd2}	PfCRT^{3D7}	PfCRT^{Ecu1110}	PfCRT^{Dd2}
+ VP	17.5 ± 1.1	9.5 ± 1.9*	6.8 ± 1.8**	21 ± 1.5	14 ± 0.9*	8.6 ± 0.2**
+ CP	9.8 ± 0.5	6.2 ± 0.3*	5.0 ± 1.1**	12 ± 0.7	7.2 ± 0.2*	8.8 ± 0.3**
+ CQ	110 ± 5.9	72 ± 3.1**	53 ± 2.8***	77 ± 5.2	62 ± 2.6**	54 ± 3.6***
+ Q_2C	0.6 ± 0.02	0.08 ± 0.007***	0.01 ± 0.002***	0.7 ± 0.02	0.06 ± 0.003***	0.02 ± 0.004***
+ SQV	50 ± 2.3	74 ± 3.9**	89 ± 3.8***	*Trans*-stimulates		

i

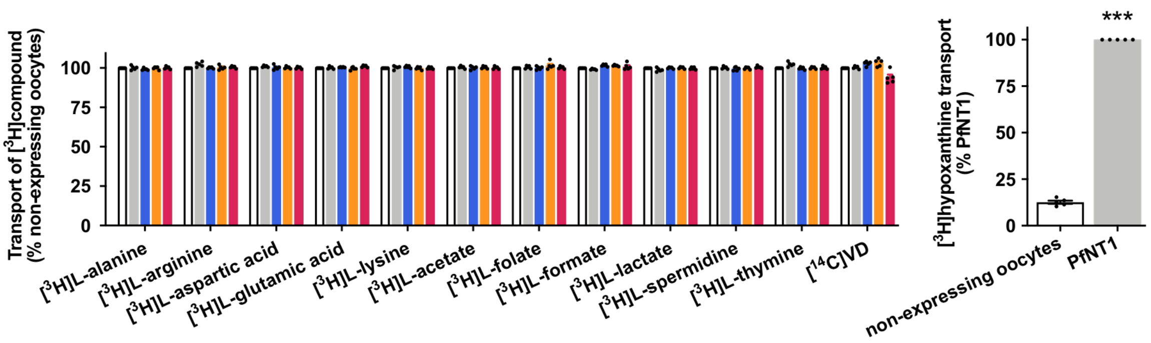

Supplementary Fig. 3. The *cis*- and *trans*-effects of known PfCRT substrates and inhibitors on [^{3}H]VF-6 transport via PfCRT in *Xenopus* oocytes. a-**c**, *Cis*-inhibition of [^{3}H]VF-6 transport via PfCRT by chlorpheniramine (CP) (**a**), CQ (**b**), and saquinavir (SQV) (**c**). **d**-**f**, *Trans*-inhibition of [^{3}H]VF-6 transport via

PfCRT by verapamil (VP) (**d**), CP (**e**), and Q_2C (**f**). **g**, Concentrations of CQ ≤ 250 µM *trans*-inhibit the PfCRT-mediated efflux of [^{3}H]VF-6 from the oocyte, whereas higher concentrations ([CQ] ≥ 500 µM) cause a modest *trans*-stimulation. **h**, IC_{50}s for the *cis*-inhibition and *trans*-inhibition of [^{3}H]VF-6 transport via PfCRT by known inhibitors and substrates of the transporter. The blue asterisks denote a significant difference from $PfCRT^{3D7}$ within a given drug treatment. **i**, Left: Various radiolabelled solutes are not transported into the oocyte by PfCRT. In the same assay, [^{3}H]CQ was taken up into the oocyte via mutant PfCRT. Right: In pairwise experiments, [^{3}H]hypoxanthine was transported into the oocyte via PfNT1. The data are the mean of n = 5 independent experiments (each yielding similar results and overlaid as individual data points in **i**) and the error is the SEM. Where not visible, the error bars fall within the symbols. The non-expressing oocyte data overlays the data obtained with oocytes expressing PfNT1 in panels **a**-**g**. The black asterisks denote a significant difference from the non-expressing control; $*P < 0.05$, $**P < 0.01$, $***P < 0.001$, ns not significant (one-way ANOVA). The source datasets are provided as a Source Data file.

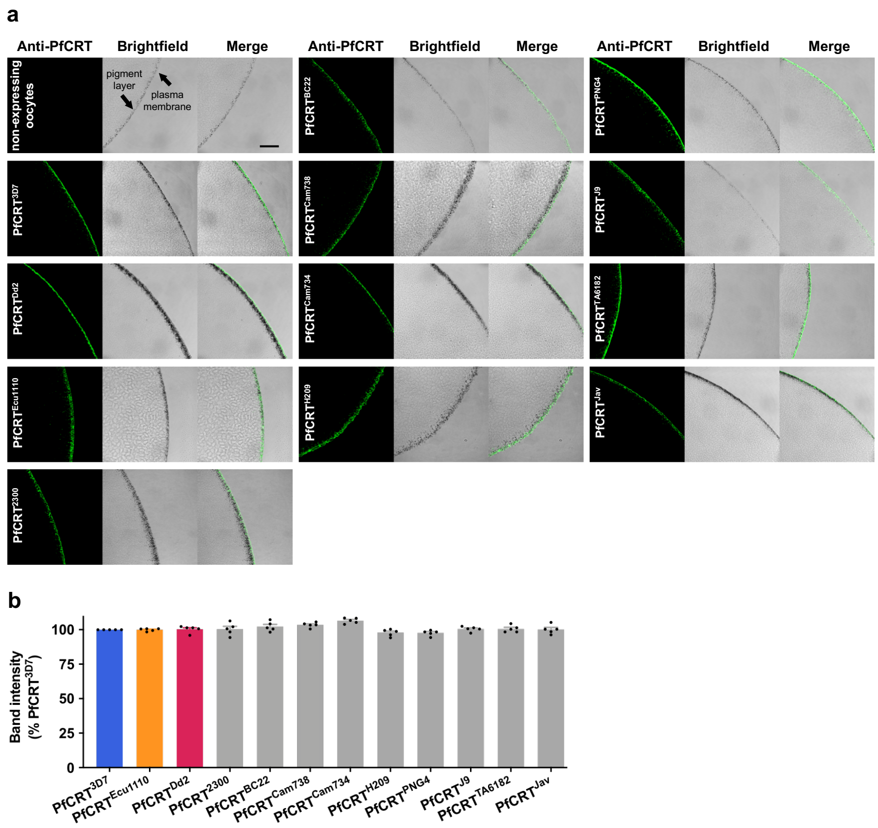

Supplementary Fig. 4. Isoforms of PfCRT localise to the plasma membrane of *Xenopus* oocytes. **a**, Immunofluorescence microscopy was used to localise various isoforms of PfCRT in the oocyte. In each case, PfCRT expression resulted in a fluorescent band external to the pigment layer, indicating that the protein is expressed in the oocyte plasma membrane. The band was not present in non-expressing oocytes. The length of the scale bar is 50 μm. **b**, The level of PfCRT protein in the oocyte membrane was semiquantified using an established western blot method. $PfCRT^{3D7}$ was included as a positive control, to which the other band intensity values were normalised. The data in panel **a** are representative of n = 2 independent experiments. The data in panel **b** are the mean of n = 5 independent experiments (each yielding similar results and overlaid as individual data points) and the error is the SEM. Where not visible, the error bars fall within the symbols. There were no significant differences in expression levels between the various PfCRT isoforms ($P > 0.05$; one-way ANOVA). Hence, all of the PfCRT variants localise to the oocyte plasma membrane and are present at similar levels in the oocyte membrane. Any differences in [^{3}H]VF-6 transport activity between these PfCRT proteins can thus be attributed to differences in their transport properties rather than variations in expression levels. The source datasets are provided as a Source Data file.

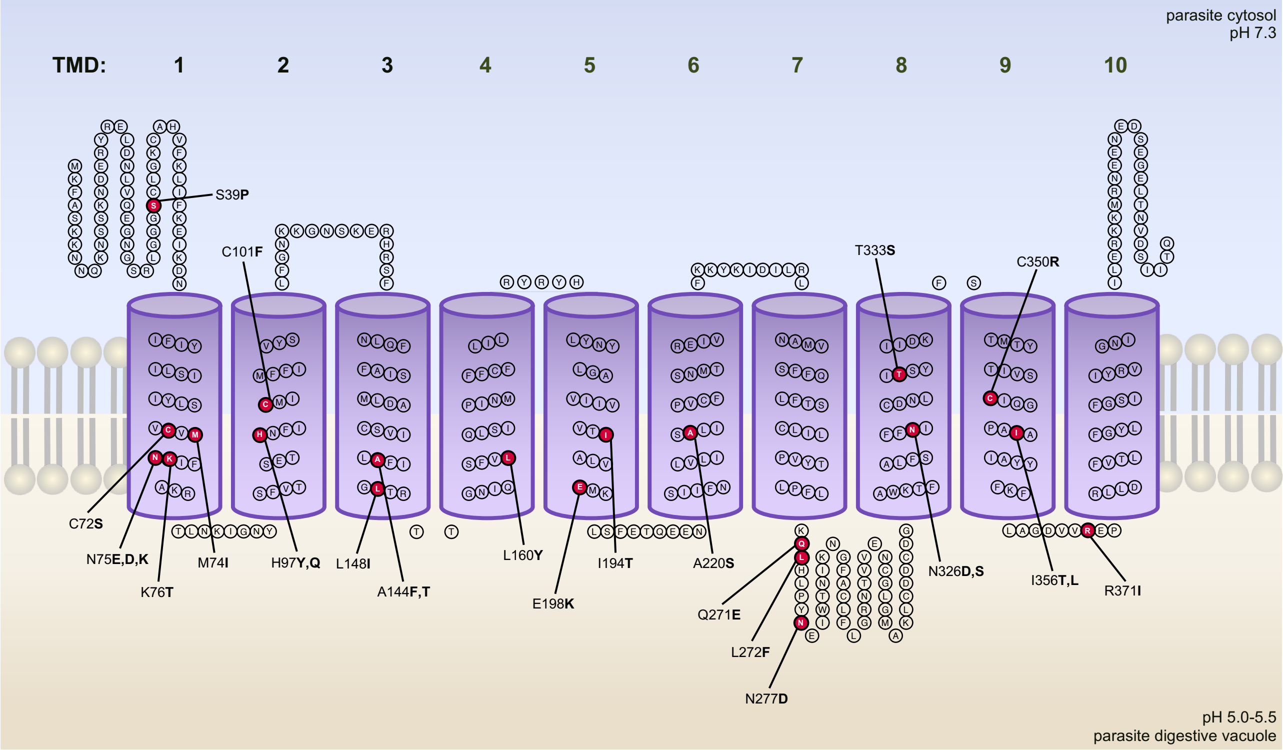

Supplementary Fig. 5. Predicted topology of PfCRT showing the mutations present in the field and laboratory-derived isoforms of PfCRT characterised in this study. PfCRT is predicted to contain 10 α-helical transmembrane domains (TMDs) and to be orientated in the digestive vacuole (DV) membrane with the N- and C-termini extending into the parasite cytosol. The positions of the mutated amino acid residues are indicated with red circles.

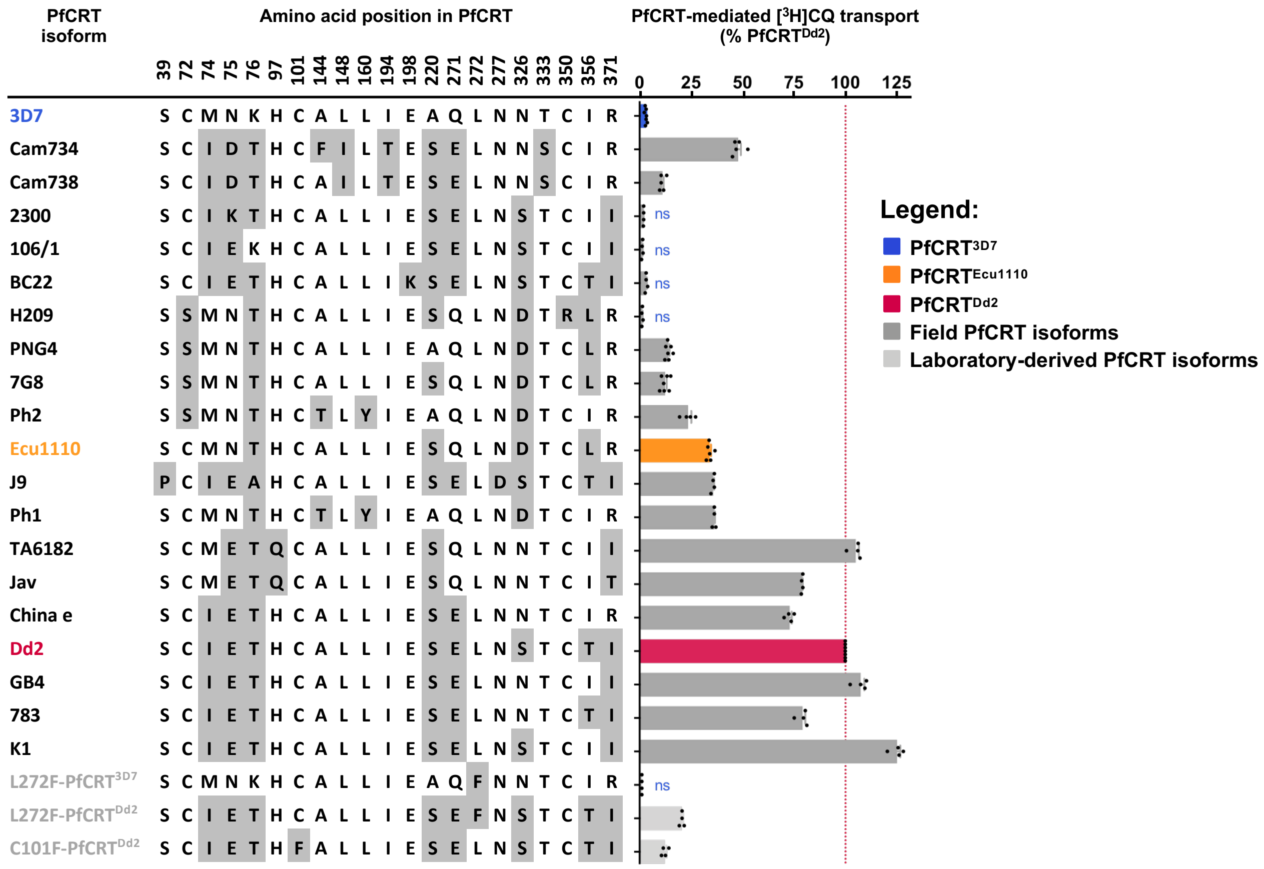

Supplementary Fig. 6. CQ transport activities of various field and laboratory-derived PfCRT isoforms in *Xenopus* oocytes. Measurements of [^{3}H]CQ uptake were undertaken with oocytes expressing a field or laboratory-derived isoform of PfCRT. The data are the mean of multiple independent experiments (each yielding similar results and overlaid as individual data points) and the error is the SEM. Where not visible, the error bars fall within the symbols. The number of independent experiments performed for each variant were as follows: PfCRT^{Cam734} and PfCRT^{Cam738}, n = 5; PfCRT^{Ecu1110}, n = 6; PfCRT^{3D7}, PfCRT^{PNG4}, PfCRT^{7G8}, and PfCRT^{Dd2} , n = 7; the remaining PfCRT isoforms, n = 4. ns denotes no significant difference from PfCRT^{3D7} (*P* > 0.05; one-way ANOVA). The source datasets are provided as a Source Data file.

a

pH	Percentages of selected compounds in their neutral and protonated forms		
	**CQ**		
	**% CQ**	**% CQH$^+$**	**% CQH$_2^{2+}$**
5	0.0000	0.0800	99.9200
5.5	0.0000	0.2525	99.7475
6	0.0000	0.7943	99.2057
7.4	0.0263	16.7393	83.2343
	**SQV**		
	**% SQV**	**% SQVH$^+$**	**% SVQH$_2^{2+}$**
5	0.0339	99.9629	0.0032
5.5	0.1070	99.8919	0.0010
6	0.3377	99.6620	0.0003
7.4	7.8438	92.1562	0.0000
	**Indinavir**		
	**% IDV**	**% IDVH$^+$**	
5	0.4181	99.5819	
5.5	1.3244	98.6756	
6	4.0848	95.9152	
7.4	51.7230	48.277	
	**Nelfinavir**		
	**% NFV**	**% NFVH$^+$**	
5	0.0660	99.9340	
5.5	0.2085	99.7915	
6	0.6564	99.3436	
7.4	14.2337	85.7663	
	**E-64**		
	**% E-64**	**% E-64H$^+$**	
5	0.0001	99.9999	
5.5	0.0002	99.9998	
6	0.0005	99.9995	
7.4	0.0135	99.9865	
	**Leupeptin**		
	**% LEU**	**% LEUH$^+$**	
5	0.0001	99.9999	
5.5	0.0002	99.9998	
6	0.0005	99.9995	
7.4	0.0135	99.9865	
	**Ac-YF-5-NH$_2$**		
	**% YF-5**	**% YF-5H$^+$**	
5	2.0465	97.9535	
5.5	6.1975	93.8025	
6	17.2822	82.7178	
7.4	83.9951	16.0049	
	**Ac-ST-7-NH$_2$**		
	**% ST-7**	**% ST-7H$^+$**	
5	2.0465	97.9535	
5.5	6.1975	93.8025	
6	17.2822	82.7178	
7.4	83.9951	16.0049	

b

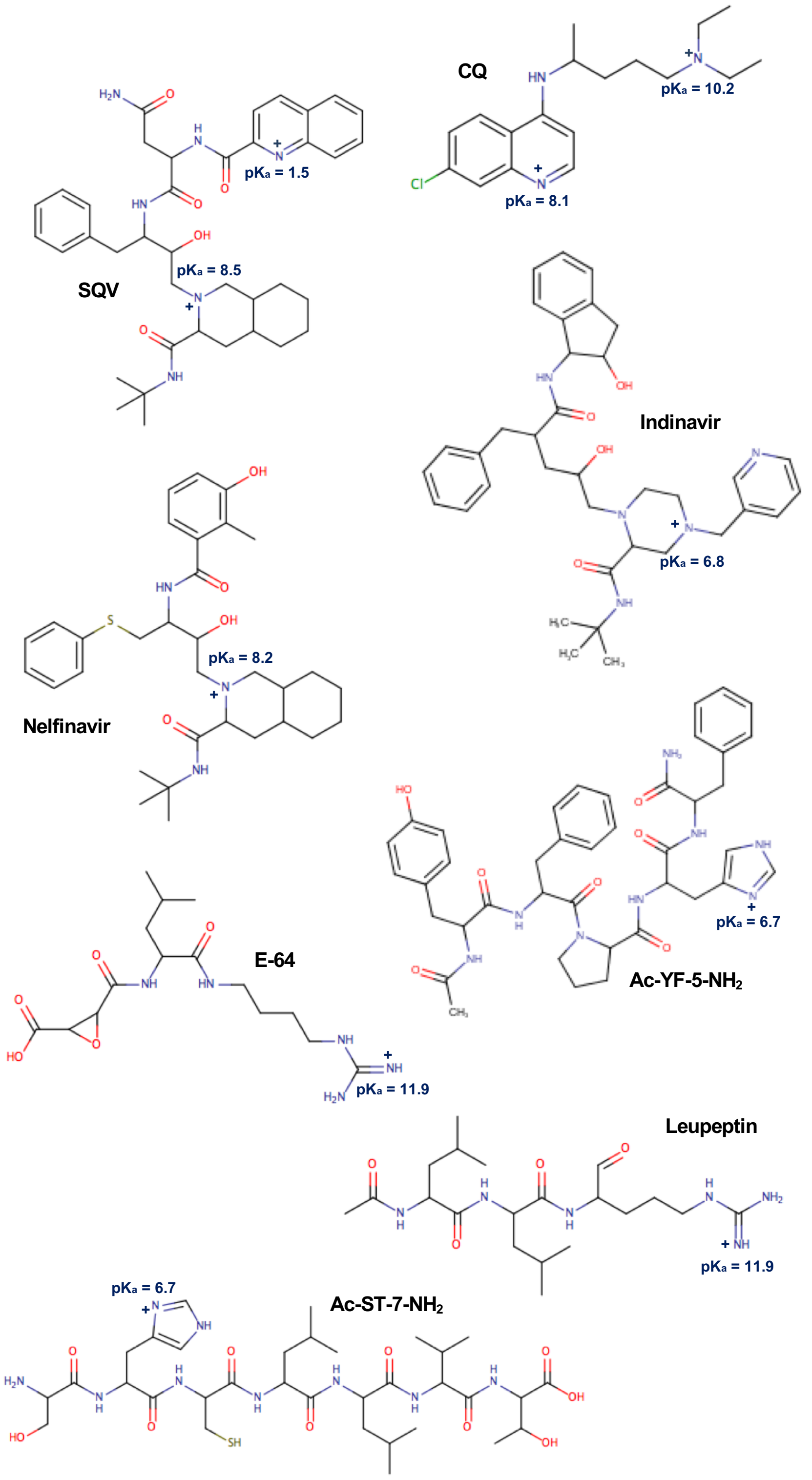

Supplementary Fig. 7. The structures of several of the peptide mimics used in this study. a, The percentages of each peptide mimic in its neutral and protonated forms in solutions of different pH. CQ is included for comparison. **b**, The protonatable nitrogen(s) within each peptide mimic. The structures and pK_a values were generated in MarvinSketch software (ChemAxon). pK_a, the negative logarithm to the base 10 of the acid dissociation constant.

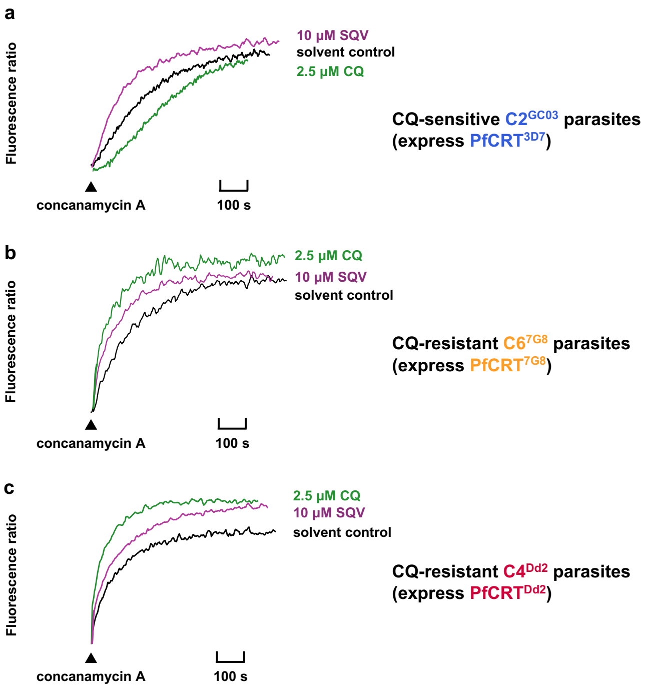

d

Experimental system	Treatment	SQV	Ac-YF-5-NH_2
		Maximum rate of peptide mimic-induced DV alkalinization (fluorescence ratio/s)	
In situ *P. falciparum* assay	$C2^{GC03}$	0.039 ± 0.002	0.018 ± 0.002
	$C6^{7G8}$	0.022 ± 0.004	0.014 ± 0.005
	$C4^{Dd2}$	0.011 ± 0.003	0.006 ± 0.0001
		App V_{max} for the *trans*-stimulatory effect of the peptide mimic (pmol/oocyte/h)	
In vitro oocyte assay	$PfCRT^{3D7}$	2.2 ± 0.5	1.2 ± 0.8
	$PfCRT^{Ecu1110}$	1.6 ± 0.2	0.4 ± 0.01
	$PfCRT^{Dd2}$	0.8 ± 0.05	0.3 ± 0.02

e

Parasite line/strain	CQ IC_{50} (nM) Control	CQ IC_{50} (nM) + 1 µM VP	Nelfinavir IC_{50} (µM) Control	Nelfinavir IC_{50} (µM) + 1 µM VP	Indinavir IC_{50} (µM) Control	Indinavir IC_{50} (µM) + 1 µM VP
$C2^{GC03}$	27 ± 1.6	26 ± 1.3	13.1 ± 0.8	4.6 ± 0.3**	4.2 ± 0.2	1.2 ± 0.8*
$C6^{7G8}$	87 ± 2.1	25 ± 0.2***	6.2 ± 0.3	1.4 ± 0.1**	1.1 ± 0.05	0.4 ± 0.02*
$C4^{Dd2}$	149 ± 4.6	26 ± 0.1***	4.1 ± 0.1	0.7 ± 0.03**	2.2 ± 0.1	0.3 ± 0.05*
3D7	25 ± 1.1	24 ± 0.9	15.3 ± 1.2	5.6 ± 0.8**	4.1 ± 0.1	2.1 ± 0.8*
7G8	94 ± 4.4	28 ± 1.6***	7.3 ± 0.6	3.1 ± 0.3*	2.5 ± 0.2	0.5 ± 0.06*
Dd2	151 ± 4.8	25 ± 1.0***	5.7 ± 0.5	0.5 ± 0.02**	1.8 ± 0.4	0.3 ± 0.08*

Supplementary Fig. 8. Peptide mimics are transported by PfCRT *in vitro* and *in situ*. a-c, Representative traces showing DV alkalinisation following the addition of concanamycin A (100 nM, indicated by the black triangle), in the presence or absence of saquinavir (SQV) or CQ, to isolated trophozoite-stage $C2^{GC03}$ (**a**), $C6^{7G8}$ (**b**), or $C4^{Dd2}$ (**c**) parasites. **d**, The apparent V_{max} values for the PfCRT-mediated transport of SQV and Ac-YF-5-NH_2 generated with (1) the parasite H^+-efflux assay or (2) the oocyte *trans*-stimulation transport assay. **e**, The antiplasmodial activities of CQ, nelfinavir, and indinavir against CQ-sensitive ($C2^{GC03}$ and 3D7) and CQ-resistant ($C6^{7G8}$, $C4^{Dd2}$, 7G8, and Dd2) parasites. The asterisks denote a significant difference between the control and 1 µM verapamil (VP) treatments within a given parasite line/strain; *$P < 0.05$, **$P < 0.01$, ***$P < 0.001$ (one-way ANOVA). The data in panels **d** and **e** are the mean of n = 5 independent experiments and the error is the SEM.

Supplementary References

1. Martin, R. E., Marchetti, R. V., Cowan, A. I., Howitt, S. M., Broer, S., & Kirk, K. Chloroquine transport via the malaria parasite's chloroquine resistance transporter. *Science.* https://doi.org/10.1126/science.1175667 **325**, 1680-1682 (2009).
2. Summers, R. L., Dave, A., Dolstra, T. J., Bellanca, S., Marchetti, R. V., Nash, M. N., Richards, S. N., Goh, V., Schenk, R. L., Stein, W. D., Kirk, K., Sanchez, C. P., Lanzer, M., & Martin, R. E. Diverse mutational pathways converge on saturable chloroquine transport via the malaria parasite's chloroquine resistance transporter. *Proc Natl Acad Sci U S A.* https://doi.org/10.1073/pnas.1322965111 **111**, E1759-1767 (2014).
3. Lehane, A. M., Hayward, R., Saliba, K. J., & Kirk, K. A verapamil-sensitive chloroquine-associated H^+ leak from the digestive vacuole in chloroquine-resistant malaria parasites. *J Cell Sci.* https://doi.org/10.1242/jcs.016758 **121**, 1624-1632 (2008).
4. Lehane, A. M., & Kirk, K. Chloroquine resistance-conferring mutations in *pfcrt* give rise to a chloroquine-associated H^+ leak from the malaria parasite's digestive vacuole. *Antimicrob Agents Chemother.* https://doi.org/10.1128/aac.00666-08 **52**, 4374-4380 (2008).
5. Lehane, A. M., & Kirk, K. Efflux of a range of antimalarial drugs and 'chloroquine resistance reversers' from the digestive vacuole in malaria parasites with mutant PfCRT. *Mol Microbiol.* https://doi.org/10.1111/j.1365-2958.2010.07272.x **77**, 1039-1051 (2010).
6. Bellanca, S., Summers, R. L., Meyrath, M., Dave, A., Nash, M. N., Dittmer, M., Sanchez, C. P., Stein, W. D., Martin, R. E., & Lanzer, M. Multiple drugs compete for transport via the *Plasmodium falciparum* chloroquine resistance transporter at distinct but interdependent sites. *J Biol Chem.* https://doi.org/10.1074/jbc.M114.614206 **289**, 36336-36351 (2014).
7. Richards, S. N., Nash, M. N., Baker, E. S., Webster, M. W., Lehane, A. M., Shafik, S. H., & Martin, R. E. Molecular mechanisms for drug hypersensitivity induced by the malaria parasite's chloroquine resistance transporter. *PLoS Pathog.* https://doi.org/10.1371/journal.ppat.1005725 **12**, e1005725 (2016).
8. Van Schalkwyk, D. A., Nash, M. N., Shafik, S. H., Summers, R. L., Lehane, A. M., Smith, P. J., & Martin, R. E. Verapamil-sensitive transport of quinacrine and methylene blue via the *Plasmodium falciparum* chloroquine resistance transporter reduces the parasite's susceptibility to these tricyclic drugs. *J Infect Dis.* https://doi.org/10.1093/infdis/jiv509 **213**, 800-810 (2016).
9. Martin, R. E., & Kirk, K. The malaria parasite's chloroquine resistance transporter is a member of the drug/metabolite transporter superfamily. *Mol Biol Evol.* https://doi.org/10.1093/molbev/msh205 **21**, 1938-1949 (2004).
10. Martin, R. E., & Kirk, K. Transport of the essential nutrient isoleucine in human erythrocytes infected with the malaria parasite *Plasmodium falciparum*. *Blood.* https://doi.org/10.1182/blood-2005-11-026963 **109**, 2217-2224 (2007).
11. Marchetti, R. V., Lehane, A. M., Shafik, S. H., Winterberg, M., Martin, R. E., & Kirk, K. A lactate and formate transporter in the intraerythrocytic malaria parasite, *Plasmodium falciparum*. *Nat Commun.* https://doi.org/10.1038/ncomms7721 **6**, 6721 (2015).
12. Hapuarachchi, S. V., Cobbold, S. A., Shafik, S. H., Dennis, A. S. M., Mcconville, M. J., Martin, R. E., Kirk, K., & Lehane, A. M. The malaria parasite's lactate transporter PfFNT is the target of antiplasmodial compounds identified in whole cell phenotypic screens. *PLoS pathogens.* https://doi.org/10.1371/journal.ppat.1006180 **13**, e1006180-e1006180 (2017).
13. Elliott, J. L., Saliba, K. J., & Kirk, K. Transport of lactate and pyruvate in the intraerythrocytic malaria parasite, *Plasmodium falciparum*. *Biochem J.* https://doi.org/10.1042/bj3550733 **355**, 733-739 (2001).
14. Carter, N. S., Ben Mamoun, C., Liu, W., Silva, E. O., Landfear, S. M., Goldberg, D. E., & Ullman, B. Isolation and functional characterization of the PfNT1 nucleoside transporter gene from *Plasmodium falciparum*. *J Biol Chem.* https://doi.org/10.1074/jbc.275.14.10683 **275**, 10683-10691 (2000).
15. Parker, M. D., Hyde, R. J., Yao, S. Y., Mcrobert, L., Cass, C. E., Young, J. D., Mcconkey, G. A., & Baldwin, S. A. Identification of a nucleoside/nucleobase transporter from *Plasmodium falciparum*, a novel target for anti-malarial chemotherapy. *Biochem J.* https://doi.org/10.1042/0264-6021:3490067 **349**, 67-75 (2000).

16. Stein, W. 1986. *Transport and diffusion across cell membranes*, New York: Academic Press.

17. Kumar, S., & Bandyopadhyay, U. Free heme toxicity and its detoxification systems in human. *Toxicol Lett.* https://doi.org/10.1016/j.toxlet.2005.03.004 **157**, 175-188 (2005).

18. Saliba, K. J., Horner, H. A., & Kirk, K. Transport and metabolism of the essential vitamin pantothenic acid in human erythrocytes infected with the malaria parasite *Plasmodium falciparum*. *J Biol Chem.* https://doi.org/10.1074/jbc.273.17.10190 **273**, 10190-10195 (1998).

19. Pulcini, S., Staines, H. M., Lee, A. H., Shafik, S. H., Bouyer, G., Moore, C. M., Daley, D. A., Hoke, M. J., Altenhofen, L. M., Painter, H. J., Mu, J., Ferguson, D. J., Llinas, M., Martin, R. E., Fidock, D. A., Cooper, R. A., & Krishna, S. Mutations in the *Plasmodium falciparum* chloroquine resistance transporter, PfCRT, enlarge the parasite's food vacuole and alter drug sensitivities. *Sci Rep.* https://doi.org/10.1038/srep14552 **5**, 14552-14567 (2015).

20. Deves, R., Chavez, P., & Boyd, C. A. Identification of a new transport system (y+L) in human erythrocytes that recognizes lysine and leucine with high affinity. *J Physiol.* https://doi.org/10.1113/jphysiol.1992.sp019275 **454**, 491-501 (1992).

21. Torrents, D., Estévez, R., Pineda, M., Fernández, E., Lloberas, J., Shi, Y. B., Zorzano, A., & Palacín, M. Identification and characterization of a membrane protein (y+L amino acid transporter-1) that associates with 4F2hc to encode the amino acid transport activity y+L. A candidate gene for lysinuric protein intolerance. *J Biol Chem.* https://doi.org/10.1074/jbc.273.49.32437 **273**, 32437-32445 (1998).

22. Bozzi, A. T., Bane, L. B., Zimanyi, C. M., & Gaudet, R. Unique structural features in an Nramp metal transporter impart substrate-specific proton cotransport and a kinetic bias to favor import. *J Gen Physiol.* https://doi.org/10.1085/jgp.201912428 **151**, 1413-1429 (2019).

23. Bazzone, A., Zabadne, A. J., Salisowski, A., Madej, M. G., & Fendler, K. A Loose Relationship: Incomplete H(+)/Sugar Coupling in the MFS Sugar Transporter GlcP. *Biophys J.* https://doi.org/10.1016/j.bpj.2017.09.038 **113**, 2736-2749 (2017).

24. Dohán, O., Portulano, C., Basquin, C., Reyna-Neyra, A., Amzel, L. M., & Carrasco, N. The Na+/I symporter (NIS) mediates electroneutral active transport of the environmental pollutant perchlorate. *Proc Natl Acad Sci U S A.* https://doi.org/10.1073/pnas.0707207104 **104**, 20250-20255 (2007).

25. Hussey, G. A., Thomas, N. E., & Henzler-Wildman, K. A. Highly coupled transport can be achieved in free-exchange transport models. *J Gen Physiol.* https://doi.org/10.1085/jgp.201912437 **152** (2020).

26. Kim, J., Tan, Y. Z., Wicht, K. J., Erramilli, S. K., Dhingra, S. K., Okombo, J., Vendome, J., Hagenah, L. M., Giacometti, S. I., Warren, A. L., Nosol, K., Roepe, P. D., Potter, C. S., Carragher, B., Kossiakoff, A. A., Quick, M., Fidock, D. A., & Mancia, F. Structure and drug resistance of the *Plasmodium falciparum* transporter PfCRT. *Nature.* https://doi.org/10.1038/s41586-019-1795-x **576**, 315-320 (2019).
