# Supplementary material for: The natural function of the malaria parasite’s chloroquine resistance transporter
Source: Nat Commun. 2020 Aug 6;11:3922. doi: 10.1038/s41467-020-17781-6 (PMC7413254; doi:10.1038/s41467-020-17781-6)
Supplement: Supplementary file 3 — Reporting Summary [file 41467_2020_17781_MOESM3_ESM.pdf]

nature research

Corresponding author(s): Rowena Martin

Last updated by author(s): Jul 10, 2020

Reporting Summary

Nature Research wishes to improve the reproducibility of the work that we publish. This form provides structure for consistency and transparency in reporting. For further information on Nature Research policies, see Authors & Referees and the Editorial Policy Checklist .

Statistics

For all statistical analyses, confirm that the following items are present in the figure legend, table legend, main text, or Methods section.

n/a	Confirmed	
☐	☒	The exact sample size (n) for each experimental group/condition, given as a discrete number and unit of measurement
☐	☒	A statement on whether measurements were taken from distinct samples or whether the same sample was measured repeatedly
☐	☒	The statistical test(s) used AND whether they are one- or two-sided<br>*Only common tests should be described solely by name; describe more complex techniques in the Methods section.*
☒	☐	A description of all covariates tested
☐	☒	A description of any assumptions or corrections, such as tests of normality and adjustment for multiple comparisons
☐	☒	A full description of the statistical parameters including central tendency (e.g. means) or other basic estimates (e.g. regression coefficient) AND variation (e.g. standard deviation) or associated estimates of uncertainty (e.g. confidence intervals)
☐	☒	For null hypothesis testing, the test statistic (e.g. F, t, r) with confidence intervals, effect sizes, degrees of freedom and P value noted<br>*Give P values as exact values whenever suitable.*
☒	☐	For Bayesian analysis, information on the choice of priors and Markov chain Monte Carlo settings
☒	☐	For hierarchical and complex designs, identification of the appropriate level for tests and full reporting of outcomes
☒	☐	Estimates of effect sizes (e.g. Cohen's d, Pearson's r), indicating how they were calculated

Our web collection on statistics for biologists contains articles on many of the points above.

Software and code

Policy information about availability of computer code

Data collection	InStat Version 3.0, Image J Version 1.8.0, SigmaPlot Windows Version 11.0, MAVEN Version 3.6, MaxQuant Version 1.5.2.8, R Statistical Software Version 3.6.3, Leica Application Suite Advanced Fluorescence software Version 3.6, MarvinSketch software version 18.10.
Data analysis	InStat Version 3.0, Image J Version 1.8.0, SigmaPlot Windows Version 11.0, MAVEN Version 3.6, MaxQuant Version 1.5.2.8, R Statistical Software Version 3.6.3, Leica Application Suite Advanced Fluorescence software Version 3.6, MarvinSketch software version 18.10.

For manuscripts utilizing custom algorithms or software that are central to the research but not yet described in published literature, software must be made available to editors/reviewers. We strongly encourage code deposition in a community repository (e.g. GitHub). See the Nature Research guidelines for submitting code & software for further information.

Data

Policy information about availability of data

All manuscripts must include a data availability statement. This statement should provide the following information, where applicable:

- Accession codes, unique identifiers, or web links for publicly available datasets
- A list of figures that have associated raw data
- A description of any restrictions on data availability

The data supporting the findings of this study are available within the paper and Supplementary Information.

Field-specific reporting

Please select the one below that is the best fit for your research. If you are not sure, read the appropriate sections before making your selection.

☒ Life sciences ☐ Behavioural & social sciences ☐ Ecological, evolutionary & environmental sciences

Life sciences study design

All studies must disclose on these points even when the disclosure is negative.

Sample size	At least three independent experiments were performed for each dataset (with the single exception of the data presented in Supplementary Fig. 4a, for which two independent experiments were performed). The number of individual experiments performed for a given dataset is reported in the figure legends. Sample size was not predetermined using statistical methods. Experiments were repeated to ensure that differences between treatments were replicated across at least three independent experiments, and to allow statistical analyses to be performed.
Data exclusions	No data were excluded from the analyses.
Replication	All experiments were replicated 3-7 times (see Figure legends for specific details for each dataset) to verify the reproducibility of the data, (with the exception of the data presented in Supplementary Fig. 4a for which two independent experiments were performed).
Randomization	Xenopus laevis oocytes were randomly selected for an experiment from the stage V-VI oocytes within a given surgery batch. This constituted n = 1. The experiment was repeated with randomly-selected stage V-VI oocytes from at least three different animals to achieve at least n = 3.
Blinding	The investigators were not blinded in this study because all of the experiments are based on objective measurements and thus the data collection and subsequent analyses are not influenced by investigator bias.

Reporting for specific materials, systems and methods

We require information from authors about some types of materials, experimental systems and methods used in many studies. Here, indicate whether each material, system or method listed is relevant to your study. If you are not sure if a list item applies to your research, read the appropriate section before selecting a response.

Materials & experimental systems

n/a	Involved in the study	
☐	☒	Antibodies
☐	☒	Eukaryotic cell lines
☒	☐	Palaeontology
☐	☒	Animals and other organisms
☐	☒	Human research participants
☒	☐	Clinical data

Methods

n/a	Involved in the study	
☒	☐	ChIP-seq
☒	☐	Flow cytometry
☒	☐	MRI-based neuroimaging

Antibodies

Antibodies used	-Custom-synthesised rabbit anti-PfCRT antibody (GenScript).<br>-Goat polyclonal anti-rabbit horesradish peroxidase conjugated (Thermo Fisher Scientific, Cat# 656120).<br>-Donkey polyclonal anti-rabbit, Alexa Fluor 488 conjugated (Thermo Fisher Scientific, Cat# A-21206).
Validation	These antibodies have previously been used and validated in Summers et al. PNAS, 2014; Richards et al. PLoS Pathog., 2016; Pulcini et al. Sci. Rep., 2015.

Eukaryotic cell lines

Policy information about cell lines

Cell line source(s)	The three transfectant Plasmodium falciparum lines were generated by Sidhu et al. Science, 2002.
Authentication	The CQ resistance phenotype of each of the transfectant Plasmodium falciparum lines was evaluated by performing cell proliferation assays, thereby yielding CQ IC50s for each parasite line. The results were verified by comparing them to previously published data (see Methods section).
Mycoplasma contamination	All cell lines tested negative for mycoplasma contamination.
Commonly misidentified lines (See ICLAC register)	None.

Animals and other organisms

Policy information about studies involving animals; ARRIVE guidelines recommended for reporting animal research

Laboratory animals	Adult (3-5 years old) female Xenopus laevis frogs.
Wild animals	This study did not involve wild animals.
Field-collected samples	This study did not involve field-collected samples.
Ethics oversight	Ethical approval of the work performed with female Xenopus laevis frogs was obtained from the Australian National University Animal Experimentation Ethics Committee (Animal Ethics Protocol Numbers R.BSB.01.10 and A2013/13) in accordance with the Australian Code of Practice for the Care and Use of Animals for Scientific Purposes (see Methods section).

Note that full information on the approval of the study protocol must also be provided in the manuscript.

Human research participants

Policy information about studies involving human research participants

Population characteristics	Human blood was used to culture the Plasmodium falciparum strains and lines used in this study. Human blood was obtained from the Australian Red Cross Blood Service.
Recruitment	Not applicable; the donors of the human blood used in this study were anonymous.
Ethics oversight	Ethical approval of the work performed with human blood was obtained from the Australian National University Human Research Ethics Committee (Human Ethics Approval Numbers 2011/266 and 2017/351; see Methods section).

Note that full information on the approval of the study protocol must also be provided in the manuscript.
