# Supplementary material for: The natural function of the malaria parasite’s chloroquine resistance transporter
Source: Nat Commun. 2020 Aug 6;11:3922. doi: 10.1038/s41467-020-17781-6 (PMC7413254; doi:10.1038/s41467-020-17781-6)
Supplement: Supplementary file 4 — Description of Additional Supplementary Files [file 41467_2020_17781_MOESM4_ESM.pdf]

Description of Additional Supplementary Files

File Name: Supplementary Data 1
Description: **Cis-inhibition and trans-stimulation of total [^{3} H]CQ transport via PfCRT in the presence of various solutes.** A solute library was screened for cis-inhibitory and trans-stimulatory activity, identifying potential substrates of PfCRT. The data are presented either as normalised to the PfCRT^{Dd2} control treatment (tab Supp Data 1a) or normalised to the oocyte control treatment (tab Supp Data 1b).

File Name: Supplementary Data 2
Description**: PfCRT-mediated cis-inhibition and trans-stimulation of [^{3} H]CQ transport in the presence of various solutes.** The PfCRT-mediated component of transport was calculated by subtracting the background accumulation of [^{3} H]CQ that occurs independently of PfCRT. The low level of [^{3} H]CQ accumulation measured in oocytes expressing PfCRT^{3D7}, which is the same level as that present in the non-expressing oocytes, is due to the simple diffusion of the neutral species of the drug into the oocyte. The data are presented either as normalised to the PfCRT^{Dd2} control treatment (tab Supp Data 2a) or normalised to the oocyte control treatment (tab Supp Data 2b).

File Name: Supplementary Data 3
Description: **Peptidomic data for C4^{Dd2}-C2^{GC03} parasites**. Host-derived peptides in erythrocytes infected with ^{C2GC03} or C4^{Dd2} parasites were quantified using tandem liquid-chromatography mass-spectrometry and the peptide levels within the CQ-resistant lines are expressed relative to those measured in the C2^{GC03} parasites.

File Name: Supplementary Data 4
Description: **Peptidomic data for C6^{7G8}-C2^{GC03} parasites.** Host-derived peptides in erythrocytes infected with ^{C2GC03} or C6^{7G8} parasites were quantified using tandem liquid-chromatography mass-spectrometry and the peptide levels within the CQ-resistant lines are expressed relative to those measured in the C2^{GC03} parasites.

File Name: Supplementary Data 5
Description: **One-sample T-test Benjamini corrected peptidomic data.** The One-sample T-test Benjamini correction was applied to the data presented in Supplementary Data 3 and Supplementary Data 4.

File Name: Supplementary Data 6
Description: **Oligonucleotide primers used in this study.** The sequences of the oligonucleotide primers, template, and mutation(s) introduced to generate the isoforms of PfCRT used in this study.

File Name: Supplementary Data 7
Description: **Buffer compositions used to clamp the oocyte membrane potential in the presence of ionophores**. The dependence of PfCRT-mediated transport on the membrane potential was examined by using Na^+ ionophore III in conjunction with the Na^+ gradients and Cl^- ionophore III in conjunction with the C^{l-} gradients listed here to clamp the membrane potential of the oocyte from -80 to +80 mV.
